# Supplementary material for: Ultrafast hole transfer mediated by polaron pairs in all-polymer photovoltaic blends
Source: Nat Commun. 2019 Jan 23;10:398. doi: 10.1038/s41467-019-08361-4 (PMC6344565; doi:10.1038/s41467-019-08361-4)
Supplement: Supplementary file 1 — Supplementary Information [file 41467_2019_8361_MOESM1_ESM.pdf]

Ultrafast hole transfer mediated by polaron pairs in all-polymer photovoltaic blends

Wang et al.

Supplementary Figures

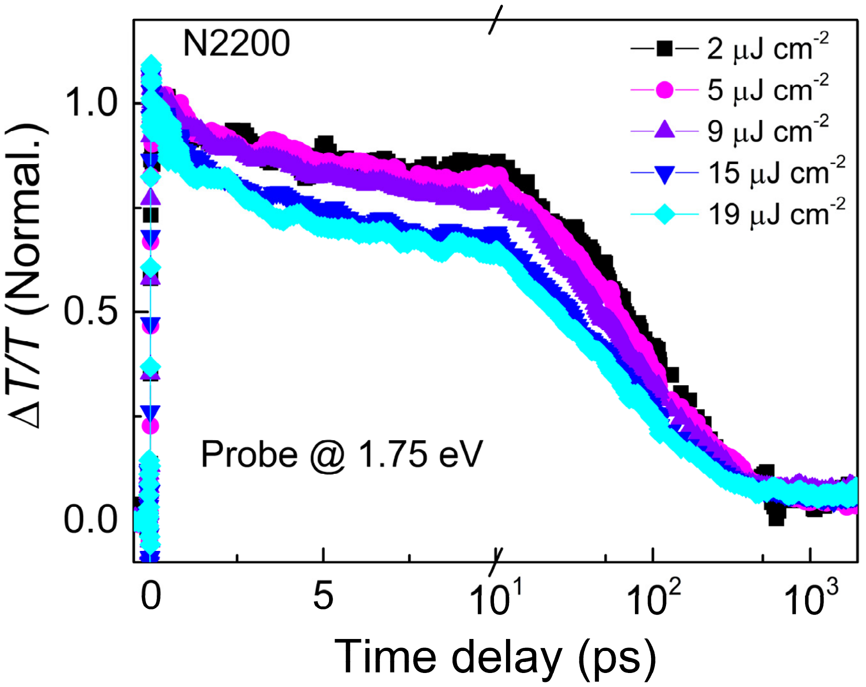

Supplementary Figure 1. Temporal evolution dynamics of the GSB signal probed at 1.75 eV recorded from a neat film of polymer N2200 under pump of different fluences.

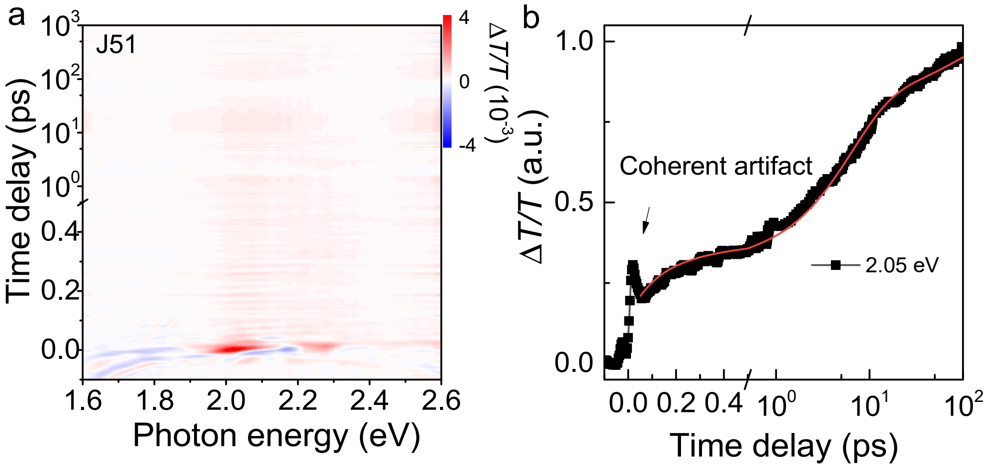

Supplementary Figure 2. (a) TA signal recorded from a neat film donor J51 with pump photon energy of 1.75 eV. The pump photon energy is below the optical gap of polymer donor J51 and therefore insufficient to excite J51. Nonetheless, a coherent response is observed in the spectral range resonant to the absorption peaks of J51, which is possibly caused by the coherent Stark effect [1]. (b) The dynamics of TA signal probed at 2.05 eV in a J51/N2200 blend film with pump photon energy of 1.75 eV. The bleach signal in the scale within the temporal resolution of the experiment is related to the coherent response.

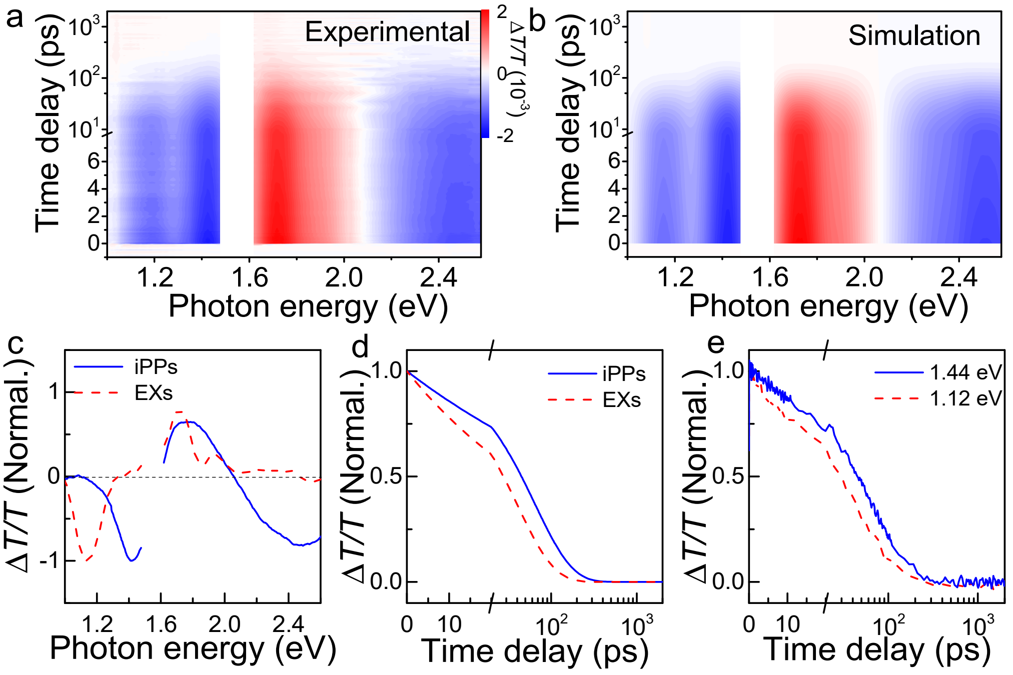

Supplementary Figure 3. Carrier dynamics in a neat film of polymer N2200. (a) TA data recorded from a neat film of polymer acceptor N2200 with pump photon energy of 1.75 eV. (b) Simulation results of TA data based on a model considering two components (of iPPs and EXs) with spectral (c) and temporal (d) characteristics using the global fitting algorithm. The spectra (c) are plotted in a scale normalized to the ESA peak. (e) The dynamics probed at 1.44 and 1.12 eV, respectively.

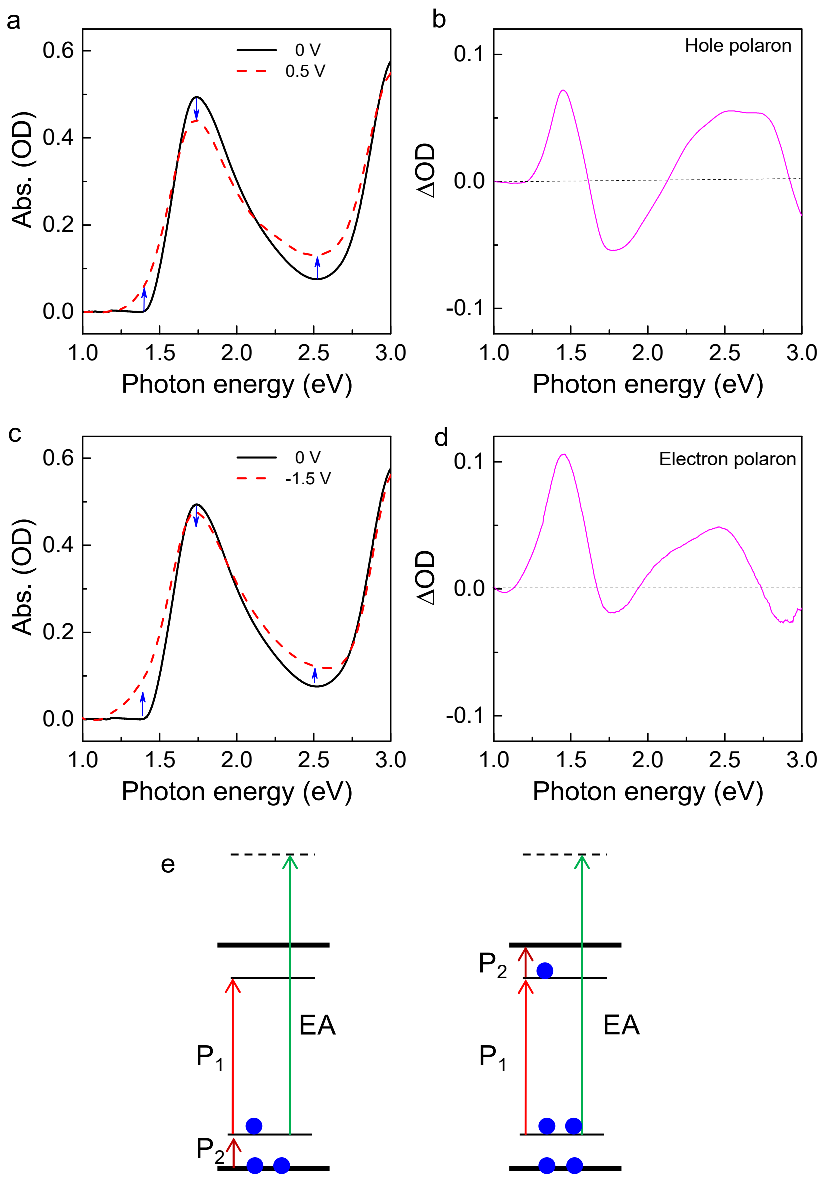

Supplementary Figure 4. In-situ spectro-electrochemistry of polymer N2200. (a) Absorption spectra of polymer N2200 recorded before and after introducing positive charges at 0.5 V. (b) The absorption change caused by positive charges showing the absorption bands of hole polarons at 1.44 and 2.4-2.6 eV. (c) Absorption spectra of polymer N2200 recorded before and after introducing negative charges at -1.5 V. (d) The absorption change caused by negative charges showing the absorption bands of electron polarons at 1.44 and 2.4-2.6 eV. (e) Diagrams of P_1, P_2 transitions of hole (left) and electron (right) polarons. EA represents a transition of polaron to higher level states. Note that the absorption spectra of hole and electron polarons are similar.

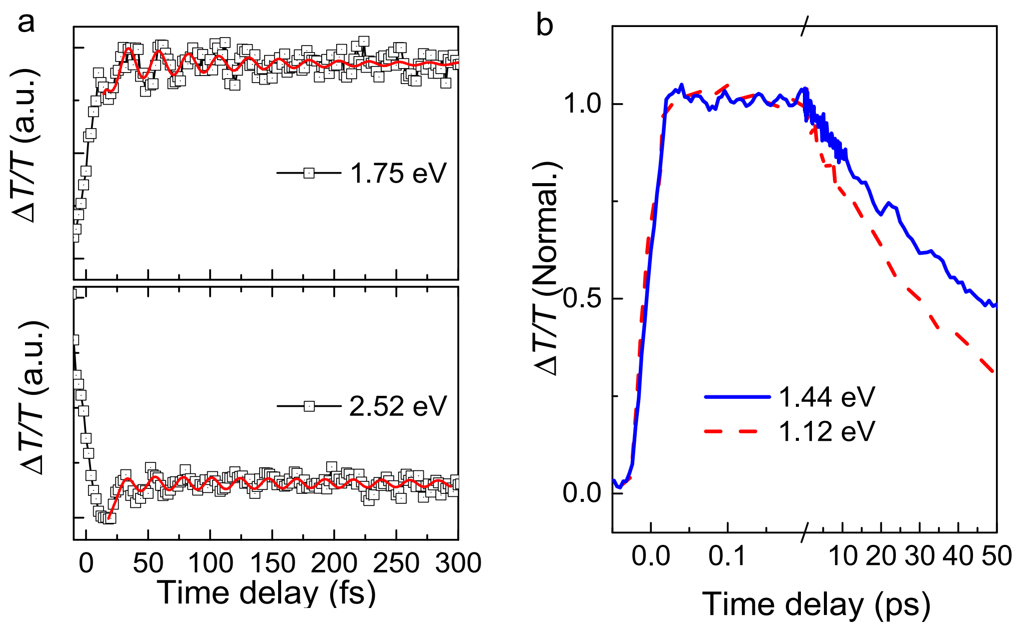

Supplementary Figure 5. Early-stage dynamics of iPPs an EXs in the neat film of polymer N2200. (a) The TA dynamics probed at 1.75 eV and 2.52 eV recorded with temporal resolution < 10 fs. (b) TA dynamics probed in the near infrared range at 1.12 eV and 1.44 eV show the simultaneous formation of iPPs and EXs in polymer N2200 within the temporal scale of instrument response.

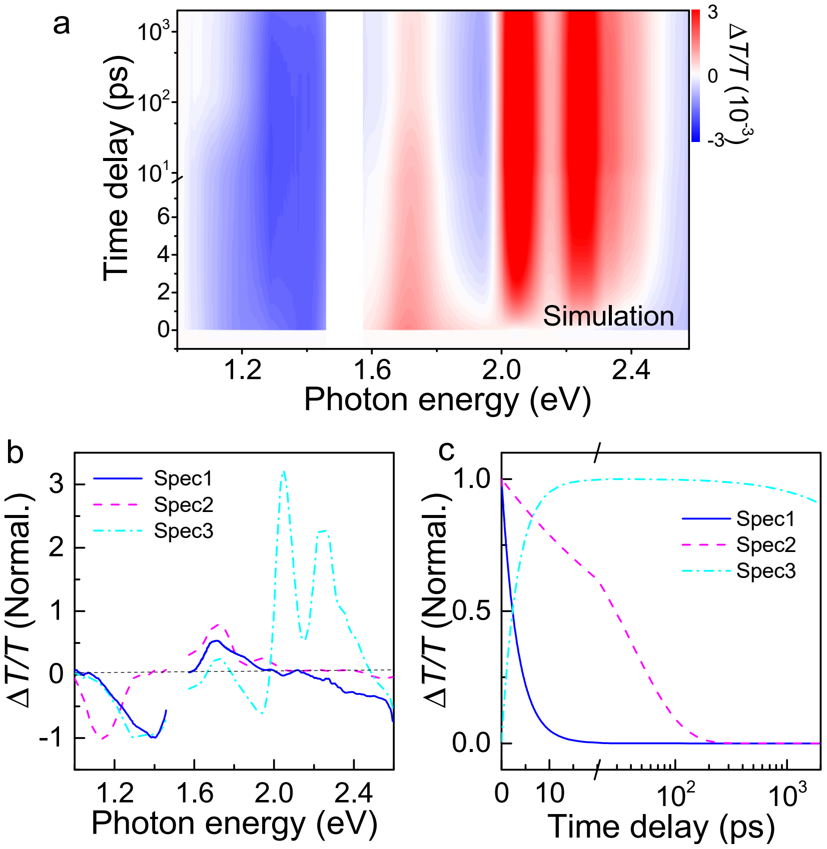

Supplementary Figure 6. Simulated TA data from the blend film. (a) Simulation can well reproduce the experimental data (Figure 2a in the text). (b) Spectral characteristics of three components: iPPs, EXs and the state resultant from hole transfer. The spectra are plotted in a scale normalized to the ESA peak. (c) The temporal characteristics of the three components.

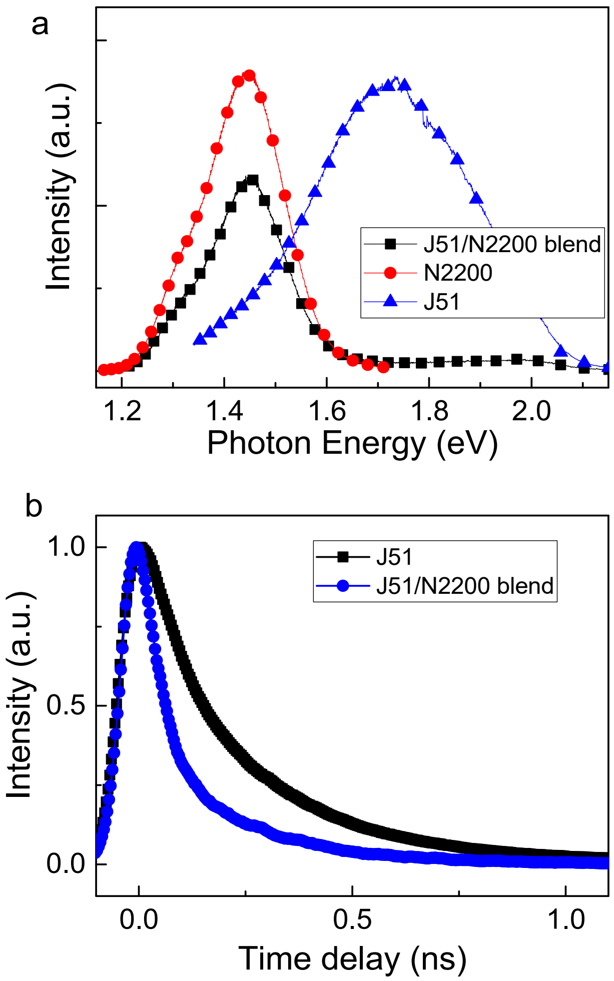

Supplementary Figure 7. (a) Fluorescence spectra recorded from the films of neat polymer donor (J51), acceptor (N2200), and their blend, respectively. (b) TRFL spectra recorded at the J51 emission peak from the neat J51 and J51/N2200 blend films, respectively.

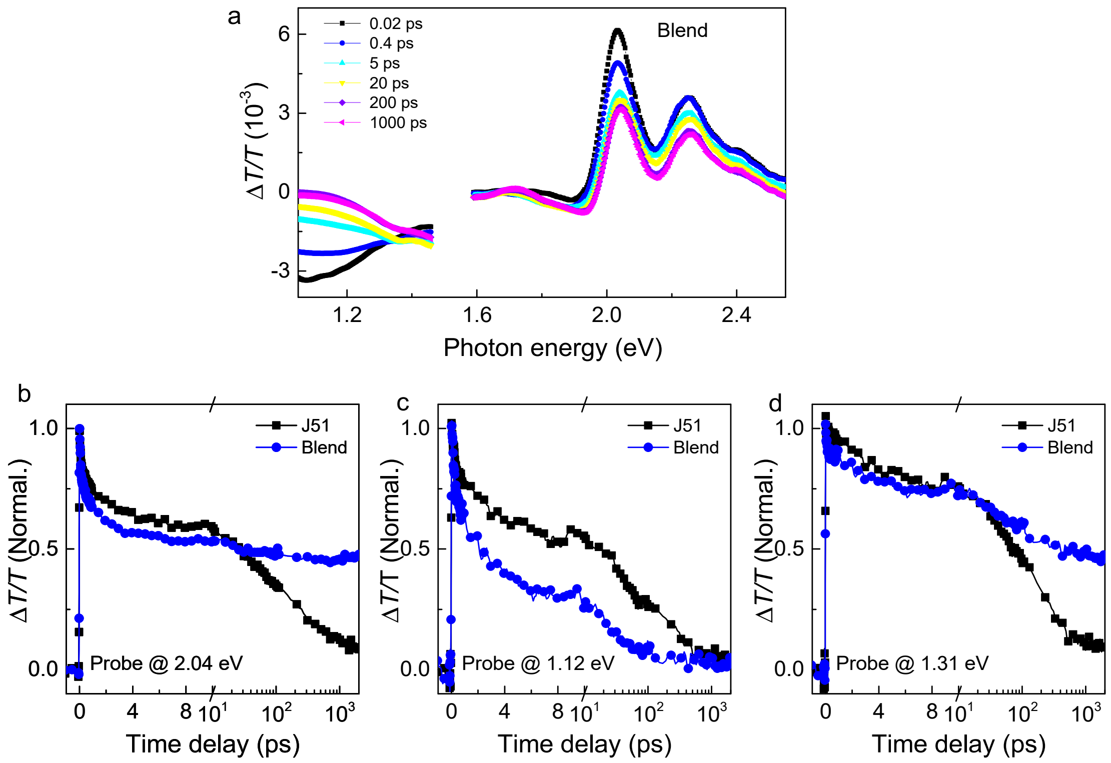

Supplementary Figure 8. Electron transfer dynamics in the J51/N2200 blend. (a) TA spectra recorded from the blend film with pump at 2.04 eV. (b-d) TA traces probed at the GSB features at 2.04 eV and two ESA features at 1.12 eV and 1.31 eV, respectively.

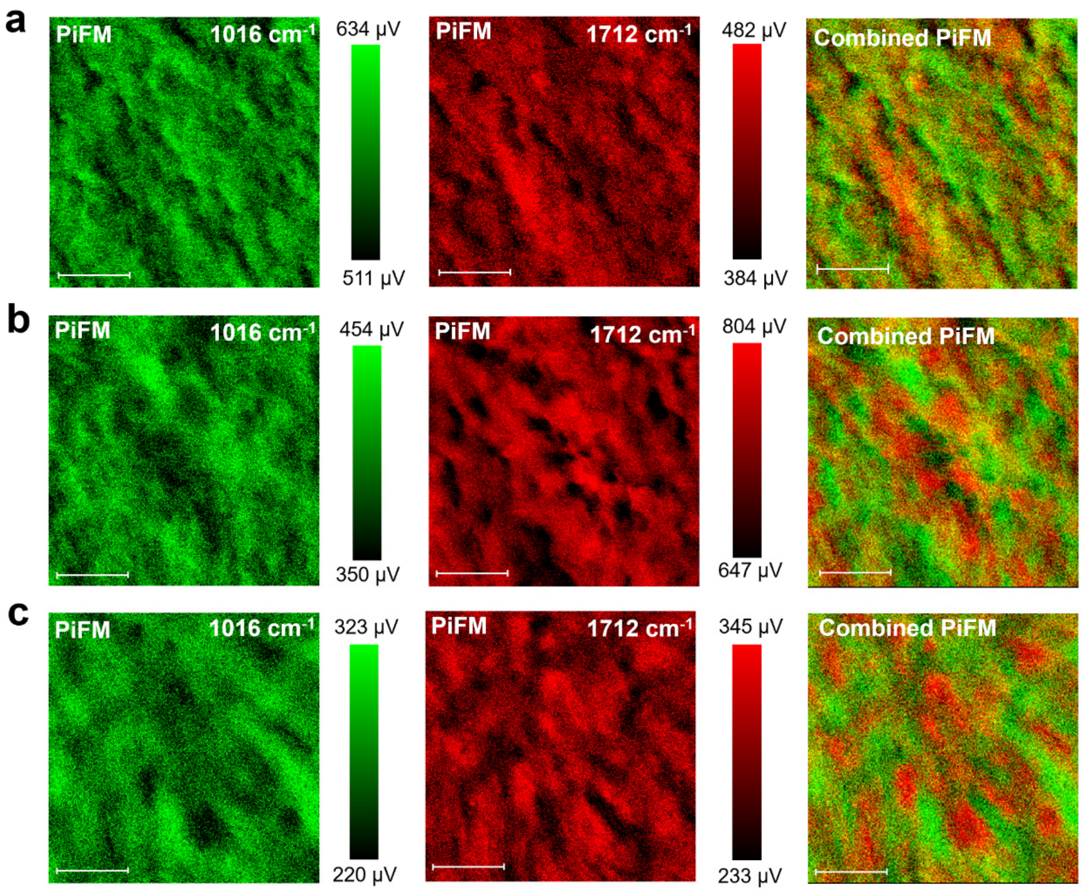

Supplementary Figure 9. Sample morphologies characterized by PiFM. PiFM images of the blend samples before (a) and after thermal annealing at 110 °C (b) and 200 °C (c), respectively. The characteristic FTIR wavelengths corresponding to the polymer donor (J51, 1061 cm^{-1}, green) and the polymer acceptor (N2200, 1712 cm^{-1}, red) are selected for better contrast of the PiFM images. The combined PiFM images show the phase separation in the blends. The scale bar is 50 nm in the figures.

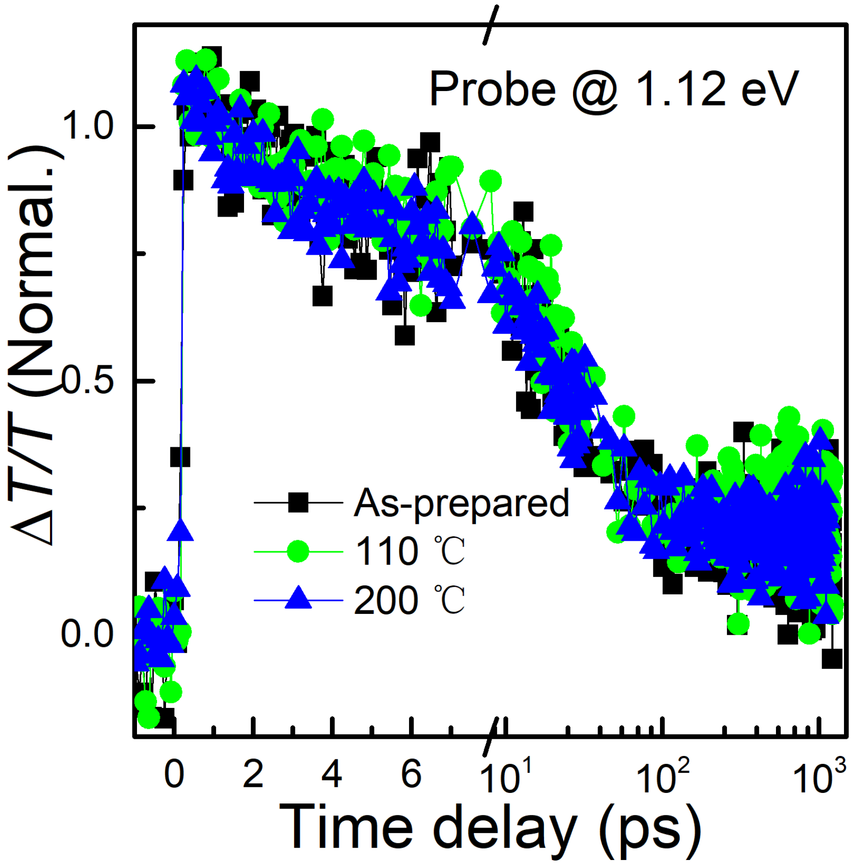

Supplementary Figure 10. TA traces probed at 1.12 eV were recorded from the blend samples before and annealed at 110 °C and 200 °C, respectively.

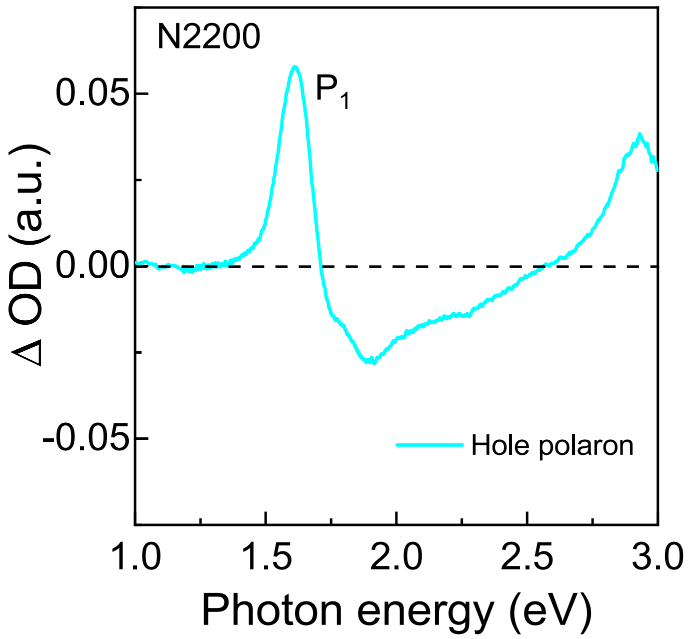

Supplementary Figure 11. Absorption change induced by chemical doping using the dopant of tris-(pentafluorophenyl)borane.

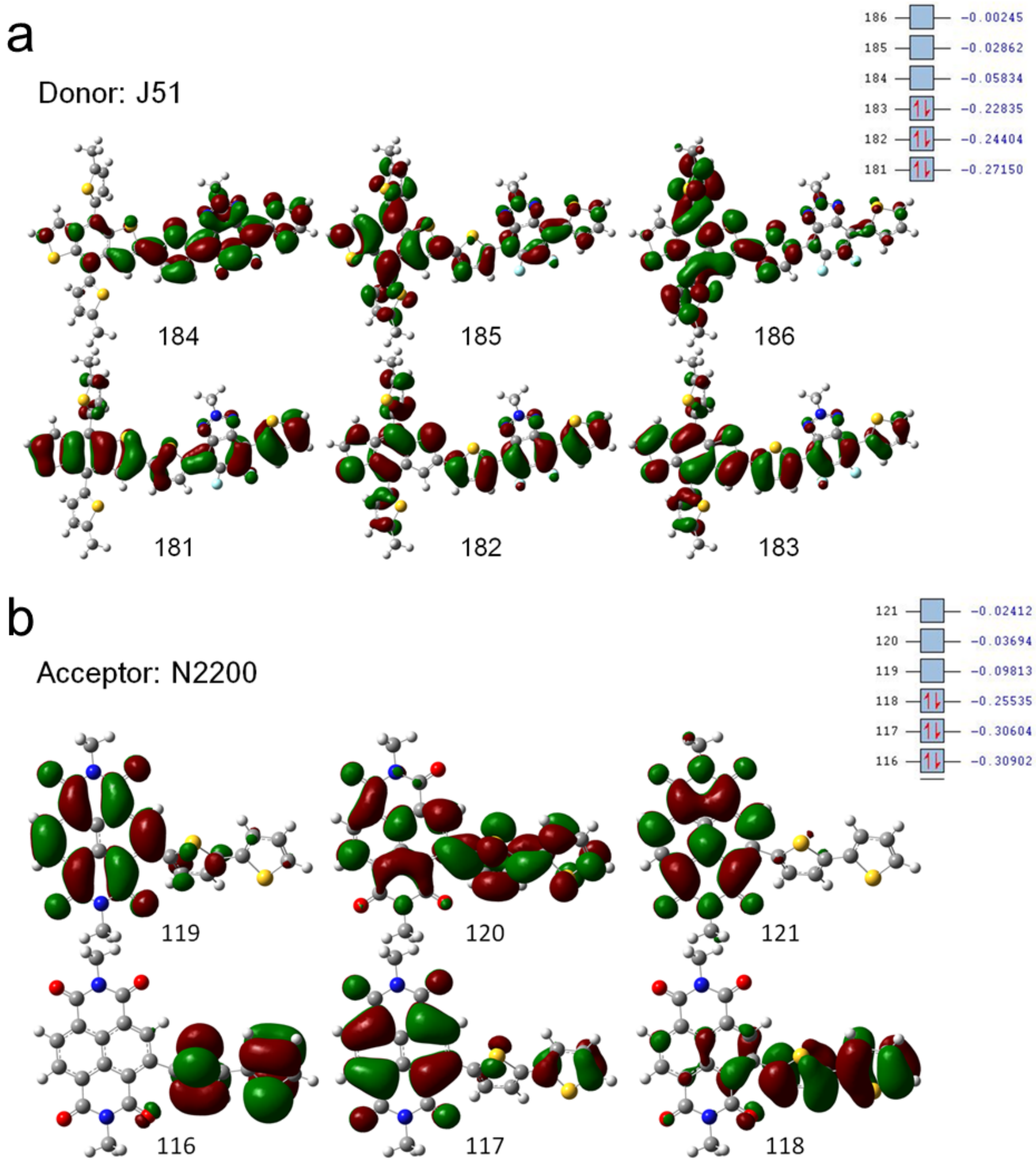

Supplementary Figure 12. Electronic structures (frontier molecular orbitals, FMO) of reduced J51 and reduced N2200 with lists of orbital energies in Hartree.

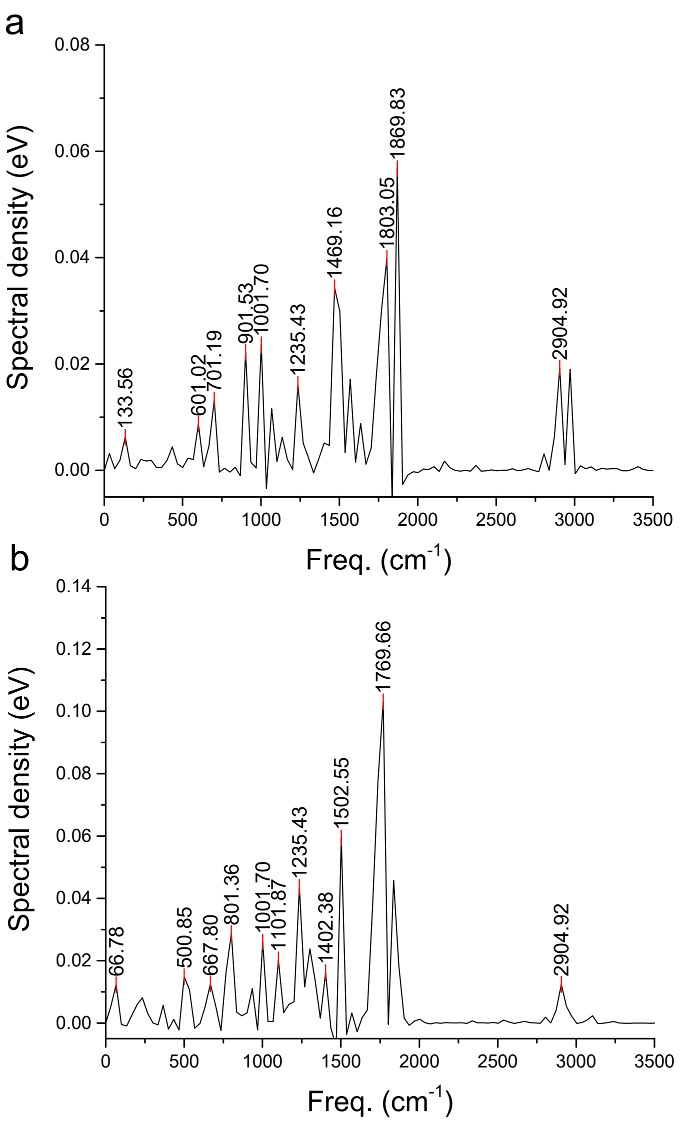

Supplementary Figure 13. Spectral densities of HOMO energies for (a) J51 and (b) N2200 with proper peak assignments.

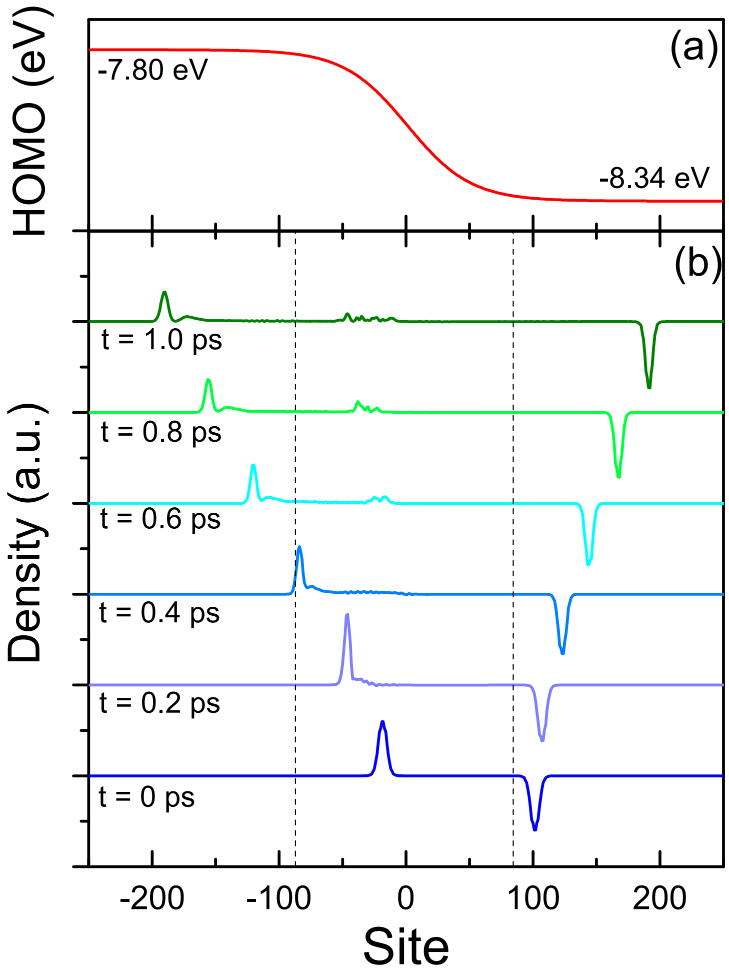

Supplementary Figure 14. Simulation of polaron pair-mediated hole transfer. (a) HOMO energy of each sites used for simulation. (b) Calculated charge density at different time delays. The curves are vertically shifted for clarity.

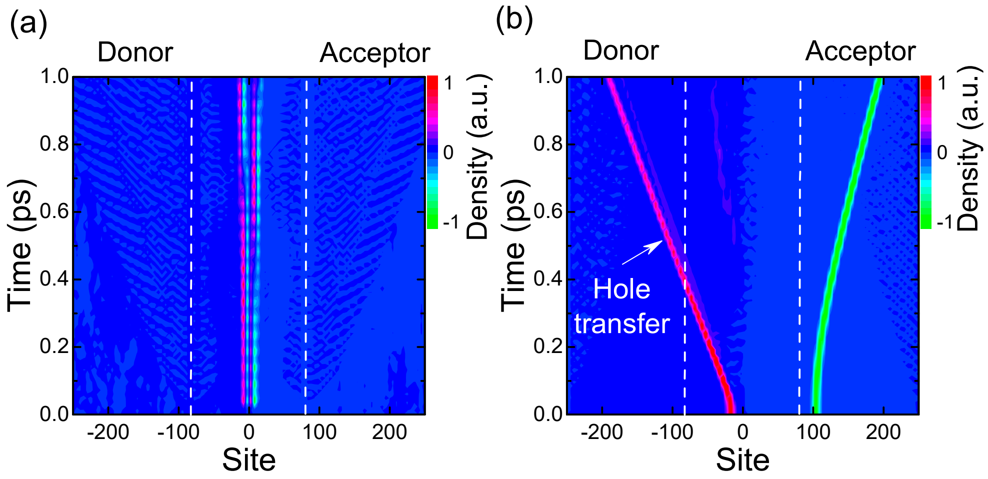

Supplementary Figure 15. Simulation results of the EX- and the iPP-mediated channels of hole transfer process. Charge density evolutions of EXs (a) and iPPs (b) at the all-polymer interface are calculated by the method of nonadiabatic dynamic simulation. The sites between the two dashed lines indicate the interface area in the numerical simulation (Supplementary Note 5).

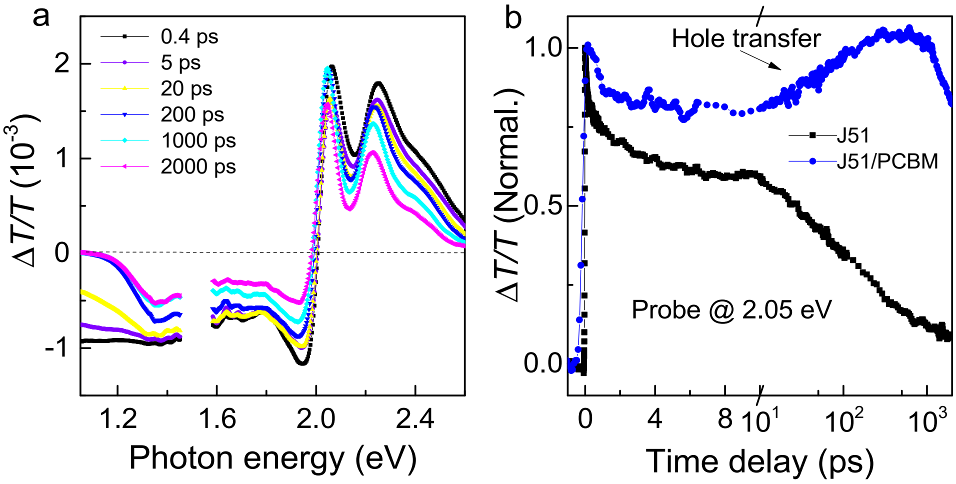

Supplementary Figure 16. TA dynamics in a J51/PCBM blend film under optical pump at 400 nm. (a) TA spectra recorded at different delay times. (b) The dynamics probed at 2.05 eV in the neat J51 and J51/PCBM blend films, respectively.

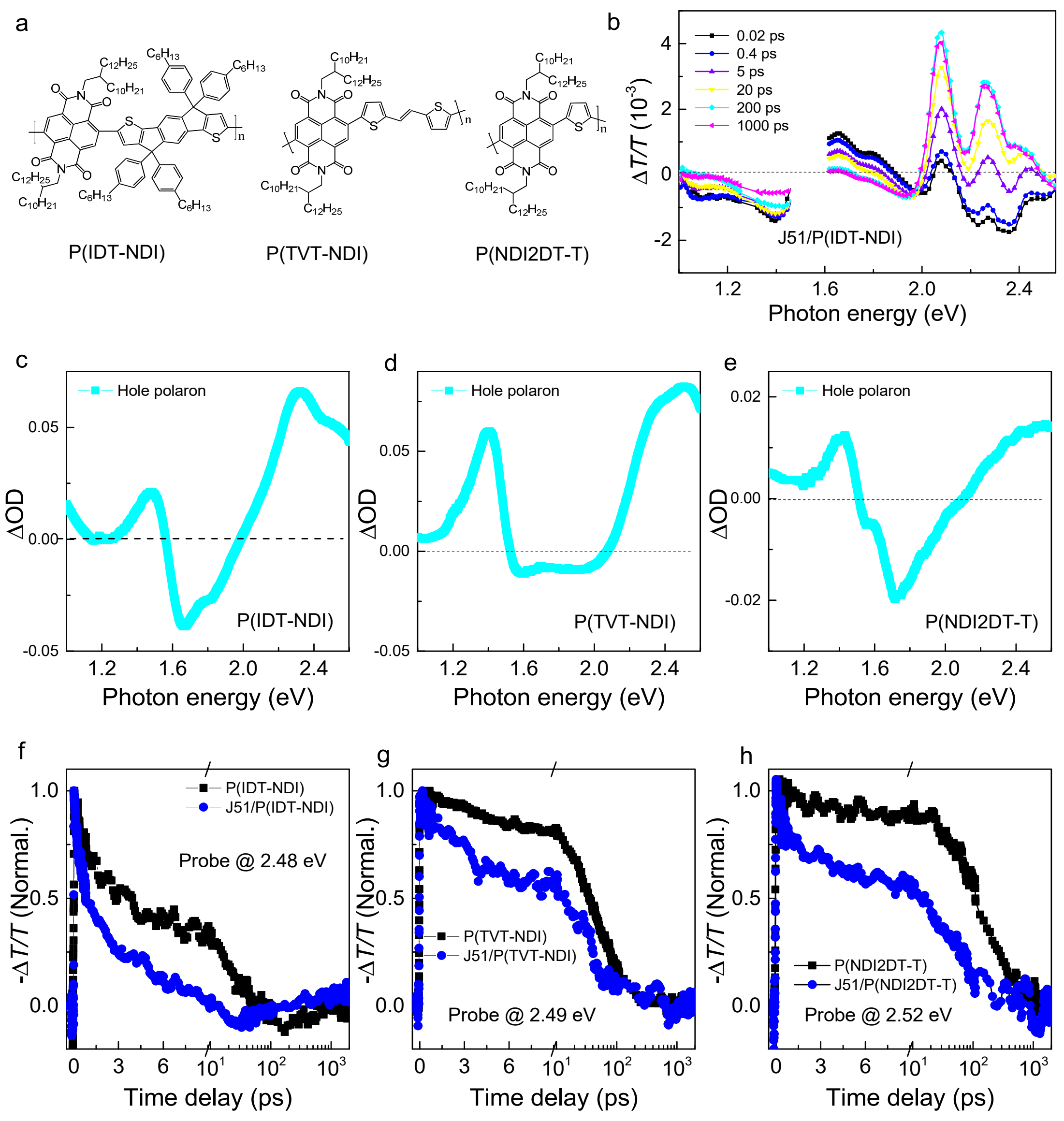

Supplementary Figure 17. iPP-mediated hole transfer process in all-polymer blends with different polymer acceptors. (a) Molecule structures of three polymer acceptors.(b) Representative TA spectra from J51:P(IDT-NDI) blend films recorded at different time delays. (c-e) Hole polaron induced absorption change in different polymer acceptors. (f-h) Dynamics of iPPs in the neat acceptor and blend films.

Supplementary Note 1. TA experiments on neat films of donor and acceptor

Supplementary Figure 1 shows the decay dynamics probed at 1.75 eV from a neat film of polymer acceptor N2200 recorded under pump with different fluences. The normalized traces are nearly independent of pump fluence in the range weaker than 5 μJ/cm^2. The results suggest that the exciton (EX) − EX annihilation effect can be neglected with pump in a weaker regime.

Supplementary Figure 2a displays the TA spectra recorded from a neat film of polymer donor J51 with pump photon energy of 1.75 eV. The pump photon energy is resonant at the absorption of N2200, but is below the absorption of J51. No signal is detected from J51 film except a coherent response at the zero time delay. The coherent response can be assigned to the Stark effect[1], *i.e.*, a shift of electronic transition energy caused by the optical field. Other than that, no TA signal is observed, confirming that the population excitation only acts on N2200 (Supplementary Figure 2a). The coherent Stark effect is also observed in the trace recorded from the blend film (Supplementary Figure 2a). Considering such signal is not caused by population occupation, we neglect it in the data analysis of hole transfer dynamics.

In N2200, the TA spectrum has included a feature of GSB, and three bands of ESA centered at different probe photon energies (Supplementary Figure 3). In polymers, primary optical excitation may create EXs or intermolecular polaron pairs (iPPs) [2-5]. To assign the features to the EXs or iPPs, we measure the absorption spectra in the samples before and after electrochemically charging. As shown in Supplementary Figure 4, absorption features at 1.45 eV and 2.4 to 2.6 eV are observed for both electron and hole polarons, which can be assigned to the P_1 and EA transitions for polarons (Supplementary Figure 4e). The P_2 transition is the mid-infrared range, exceeding the spectral coverage in our measurements. In this case, we can assign the ESA features at 1.45 eV and 2.4 to 2.6 eV to iPPs. The ESA feature at 1.12 eV is then assigned to EXs.

The spatial separation between the electron and hole for an exciton is much shorter than that for an iPP, so that the recombination rate of an exciton is generally faster than that of an iPP [2]. The signal at 1.12 eV decays faster than the signal probed at 1.45 eV (Figure 3b in manuscript), in consistence with the behaviors of EXs and iPPs,

respectively. To further confirm the assignments, we use the global fitting algorithm (Supplementary Methods) to simulate the TA experimental data from N2200 film by considering two recovery components. The simulation can well reproduce the experimental data (Supplementary Figure 3) considering two components of iPPs and EXs with spectral and temporal characteristics shown in Supplementary Figure 3c and 3d.

Supplementary Figure 5 shows early-stage dynamics probed at the featured photon energies with a higher temporal resolution. The onset of ESA signal of iPPs probed at 2.52 eV is comparable to that of GSB probed at 1.75 eV (Supplementary Figure 5a). These results suggest the simultaneous formation of iPPs and EXs, which is also confirmed by signals probed at 1.44 eV and 1.12 eV (Supplementary Figure 5b). A recent work suggested the simultaneous formation of iPPs and EXs may arise from coherent vibronic coupling [6]. The coherent scenario may be responsible for the generation of iPPs in polymer N2200 as supported by some oscillatory behaviors identified in the temporal dynamic traces (Supplementary Figure 5a).

Supplementary Note 2. Fluorescence experiments

In general, fluorescence emission is mainly contributed by the radiative recombination from photo-excited EXs. We measure the fluorescence emission spectra from the blend and the neat films to check the dynamics of EXs during interfacial charge transfer. The emission spectra from neat films of donor (J51) and acceptor (N2200) are centered at 1.75 eV and 1.45 eV, respectively (Supplementary Figure 7a). In the blend film, the donor emission is strongly quenched, suggesting that the EXs in the donor largely dissociate during electron transfer in the blend film. The quenching of acceptor emission is insignificant, confirming the spectator role of EXs in acceptor during hole transfer process in the blend films. The result is consistent with the TRFL kinetics (Figure 4 in the text).

Sample heterogeneity may cause some potential issues during TRFL analysis. We carefully evaluate the quantum efficiency of the samples using an integrated sphere. Upon 405 nm excitation, the quantum yields of fluorescence emission from N2200 are

estimated to be about 1% and 0.35% in the neat acceptor and blend films. Considering one-third of the light at 405 nm is absorbed by N2200 in the measured blend film, the obtained emission efficiencies strongly suggest that the emission dynamics of N2200 are similar in the neat acceptor N2200 and blend films, which validates the analysis based on TRFL traces in the manuscript. Moreover, TRFL traces of fluorescence emission from polymer J51 in the neat donor and blend films show clear evidence of emission quench induced by electron transfer. In other words, EX-initiated hole transfer, if there is, should be captured by TRFL spectroscopy. In addition, the decay dynamics of EX emission is longer than that characterized by TA spectroscopy, which is caused by the limited temporal resolution of TRFL measurement and different states contributed to TA and fluorescence signals.

Supplementary Note 3. Electron transfer process in J51/N2200

Electron transfer contributes significantly to the device performance. We perform TA experiments to study the dynamics of electron transfer in the J51/N2200 blend film. Supplementary Figure 8 shows the TA spectra recorded from the blend film at different time delays. Due to the overlap of the donor and acceptor absorption, it is challenging to exclusively assign the origin of different spectral features. Nonetheless, electron transfer is manifested in the early-stage dynamics where the GSB and ESA features of the donor decay much faster in the blend film in comparison with those in the neat acceptor films (Supplementary Figure 8b-d). Of particular importance, the lifetime of fluorescence decay of donor emission is significantly shortened in the blend in comparison with the decay in the neat donor (Supplementary Figure 7b). The results clearly indicate that the photo-excited EXs are largely involved in the electron transfer process which is quite different from that in the case of hole transfer.

Supplementary Note 4. Quantification of the generation yield of iPPs

To quantify the yield of iPP generation, the chemical doping approach[7] has been adopted (Supplementary Methods). The doping induced absorption can be acquired by subtracting the absorption spectrum of the pure N2200 solution from that of the doped-

N2200 solution, which is plotted in Supplementary Figure 11. The spectral feature of polaron absorption derived from chemical doping approach show a slight blueshift to that derived from spectro-electrochemistry measurements, which is induced by absorption differences of N2200 in the forms of solution and film. The infrared absorption peak can be assigned to P_1 band of the doping induced hole polarons [5, 7] which is further to calculate the polaron cross-section. The molar absorption coefficient ε_{P_1} of positively charged polarons at P_1 band in polymer N2200 can be calculated as:

$$\varepsilon_{P_1} = \frac{OD_{P_1}}{c_{dop} \cdot x} = \frac{0.058}{3.8\times10^{-6} mol/L \cdot 0.2cm} = 7.6\times10^{4} mol^{-1} \cdot L \cdot cm^{-1} \quad (1)$$

The cross section σ_P^+ can be calculated as

$$\sigma_P^+ = \ln(10) \cdot \frac{\varepsilon_{P_1}}{N_A} = 2.3 \cdot \frac{7.6\times10^{4} mol^{-1} \cdot L \cdot cm^{-1}}{6.02\times10^{23}} = 2.9\times10^{-16} cm^2 \quad (2)$$

In general, the difference of absorption cross sections between electron and hole is insignificant in conjugated polymers [7]. For simplicity, we assume the electron and hole polarons having same cross sections ($\sigma_P^- = \sigma_P^+$) in calculating the number of PPs. With these values, the generation yield of iPPs in different samples can be quantitatively evaluated. In the pristine film of N2200, the generation yield is estimated to be ~ 36%.

Supplementary Note 5. Theoretical simulation

The SSH theory is the benchmark for understanding the elementary excitations in polymers [8]. By combing the SSH theory with the nonadiabatic method, the dynamics of EXs and polarons in the polymer chains can be well sketched. It is worth noting that, in the incoherent hopping scenario in analogy to its inorganic counterpart, the electron and hole are treated as classical particles hopping among molecules and the binding energy between them solely originates from the Coulomb attraction. According to the SSH theory, however, the binding energy stems from both the Coulomb attraction and self-trapping effect induced by the vibronic couplings which is unique and significant in organic molecules.

For simulation, we consider reduced structures of J51 and N2200 by removing the

alkyl chains from the conjugated systems. The electronic structures are calculated by applying density functional theory (DFT) with M06-2X/cc-pVDZ level using Gaussian 09 (D.01) package [9]. The frontier molecular orbitals (FMO) of these two reduced polymer units are displayed in Supplementary Figure 12. The majority electron density is found to reside on the unit of thiophene. In order to build a model for mimicking the vibronic dynamics in the J51/N2200 blend, we map the polymer chain onto a one-dimensional lattice with sites on the lattice being thiophene unit.

To this end, we consider the celebrated SSH model to study the iPPs and EXs in the polymer chains [8, 10, 11]. The Hamiltonian is written as

$$H = H_{\mathrm{E}} + H_{\mathrm{L}}, \tag{3}$$

where H_{E} is the Hamiltonian for the electrons with the form being

$$H_{\mathrm{E}} = -\sum_{j,\sigma} \varepsilon_j |j,\sigma\rangle\langle j,\sigma| + [t_0 - \alpha(u_{j+1} - u_j)](|j+1,\sigma\rangle\langle j,\sigma| + \mathrm{h.c.}), \tag{4}$$

where j denotes the site index for the thiophene unit and σ for the spin index, ε_j the on-site electronic energy, t_0 the hopping integral, α the vibronic coupling strength and u_j the displacement of the j-th site. The many-body interaction between electrons is neglected since the separation distance between positive and negative charges in the iPP state is relatively large. H_{L} is the Hamiltonian of lattice which reads

$$H_{\mathrm{L}} = \frac{K}{2}\sum_j (u_{j+1} - u_j)^2 + \frac{M}{2}\sum_j \dot{u}_j^2, \tag{5}$$

where K is the elastic constant and M is the mass of the thiophene. In our computations, we take 480 sites on the lattice with 240 sites denoting for J51 ($j < 0$) and the other 240 for N2200 ($j > 0$). The 160 sites ($-80 \leqslant j \leqslant 80$) in the middle of the lattice represent the interfacial regime. The on-site energy ε_j is thus set to -7.80eV for $j < -80$ (the HOMO energy of J51) and -8.34eV for $j > 80$ (the HOMO energy of N2200), and in between the on-site energy slowly varies following a hyperbolic tangent function. t_0 is calculated to be 0.09eV for both molecules. α and K can be obtained by taking the vibrational spectrum into account. Supplementary Figure 13 displays the spectral

densities of HOMO energies for both J51 and N2200. We take 1769 cm^{-1} as the primary bare optical phonon mode ω_Q, so that K can be calculated by $K = M\omega_Q^2/4$ =231eV/Å^2. The reorganization energy calculated from the spectrum is 0.018eV [12], so that α is determined to be 3.4 eV/Å as we have calculated that the average $|u_{j+1} - u_j|$ in equilibrium is 0.00545 Å.

The nonadiabatic dynamics simulation method is employed to calculate the dynamics of the EXs and polarons in polymer chains [13, 14]. In detail, the temporal evolution of the lattice site is expressed as

$$M\ddot{u}_j(t) = K\lfloor u_{j+1}(t) + u_{j-1}(t) - 2u_j(t) \rfloor + 2\alpha \cdot \mathrm{Re}\lfloor \rho_{j,j-1}(t) - \rho_{j+1,j}(t) \rfloor - M\eta\dot{u}_j(t), \quad (6)$$

with η being the damping coefficient. Herein, $\rho_{j,j-1}$ is the relevant element of the electronic density matrix which is defined as $\rho_{j,j-1} = \sum_k f_k \varphi_{j,k}^* \varphi_{j-1,k}$ with $\varphi_{j,k}$ being the wave function of k-th orbit at j-th site whose temporal evolution follows the standard Schrödinger equation, and f_k being the occupation coefficient, namely f_k taking 0, 1 and 2 for empty, single-occupied and double-occupied orbits, respectively. For calculating the dynamics of EX, f_k is taken to be 1 for both HOMO and LUMO; For calculating the dynamics of polaron, f_k is initially taken to be 1 for HOMO and 0 for LUMO to obtain the lattice deformation of a single polaron. Afterwards, we adopt the u_j of the polaron to calculate the second polaron and then change f_k of LUMO to be 1 to calculate the following evolution. To eliminate the influence of other elementary excitations, such as the breathers, we have to deduct the dynamics of EXs and polarons with the configuration of the ground state (i.e., f_k is set to be 2 for HOMO and 0 for LUMO).

In some sense, the simulating results shown in the main text are counterintuitive. In our model, the configurations of f_k for both EX and PP are the same during the time evolution. The binding energy of the EX is underestimated if compared with that in the realistic case. In this situation, the EX is still difficult to be dissociated due to the strong

binding energy stemming from the self-trapping effect induced by the lattice deformation. On the contrary, the PP state is more mobile producing very efficient charge separation (Supplementary Figure 14). The equivalent binding energies for EXs and iPPs can be calculated to be in the order of 500 meV and 30 meV in this work, which are comparable to the calculated values in literatures.[15] It is also worth noting that, since the existence of iPP state demands relatively long conjugation length (e.g. 10 nm), the quantum yield of iPP state is however much lower than that of EX. J51 and N2200 do not seem to suffer from this problem so that they can act as high-efficiency materials for charge generation.

Supplementary Note 6. Hole transfer dynamics in different OPV blends.

Hole transfer also exists in the polymer/fullerene blends[16] although its contribution to device performance is insignificant. Unlike the polymer N2200, EXs are the primary forms of the excited states in small-molecule acceptor PCBM. In the blend of J51/PCBM, hole transfer is primarily triggered by EXs, which is different from the iPP-mediated hole transfer process in the all-polymer J51/N2200 blend. We have performed TA experiments to the interfacial hole dynamics in a blend film of J51/PCBM. Supplementary Figure 16 displays the TA spectra recorded from the blend J51/PCBM film. Due to spectral overlap, the pump wavelength of 400 nm is selected to reduce the direct excitation of J51. We observe a feature of delayed rise of bleach signal in the J51 sample (Supplementary Figure 16b) which is probably related to hole transfer. The rate of hole transfer is much slower due to energy mismatch of HOMO levels.

To draw a general conclusion, we have studied more blend systems using the same polymer donor J51 and three different polymer acceptors of naphthalene diimide-based copolymers, i.e., P(IDT-NDI), J51/P(TVT-NDI) and J51/P(NDI2DT-T) [17-20]. The molecule structures are shown in Supplementary Figure 17a. The dynamics of hole transfer in the blends are similar to that in the J51/N2200 blend. Representatively, we show the TA spectra recorded from the blend of J51/P(IDT-NDI) in Supplementary Figure 17b. The polaron absorption of the three acceptors are characterized by the spectro-electrochemical measurements (Supplementary Figure 17c-e). In all the three

systems, the ESA features of iPPs decay much faster in the blends than in the neat acceptor films (Supplementary Figure 17f-h), indicating the presence of iPP-mediated hole transfer process in all the three systems.

Supplementary Methods

Device fabrication and characterization. The photovoltaic devices used in the current study were fabricated with the polymer J51 [21] as the donor and the polymer N2200 [22] as the acceptor with a configuration of ITO (indium tin oxide)/PEDOT:PSS (poly−(3,4−ethylenedioxythiophene): poly(styrene sulfonate)) /J51:N2200 (2:1, w/w) /PDINO (perylene diimide functionalized with amino *N*-oxide) /Al [23]. The anode buffer layer of PEDOT:PSS (40 nm) was deposited through spin−coating on a pre−cleaned ITO−coated glass from a PEDOT: PSS aqueous solution (Baytron P VP AI 4083 from H. C. Starck) at 2000 rpm and dried subsequently at 150 °C for 15 min in air. Then the device was transferred to a nitrogen glove box, where the active layer (120 nm) was formed by spin-coating the polymer blend with a total blend polymer concentration of 12 mg mL^{-1} in chloroform, followed by thermal annealing at 100 °C for 10 min. 1,8-Diiodooctane (DIO) with 1% volume ratio was added to the chloroform solutions and stirred before use. Then methanol solution of PDINO at a concentration of 1.0 mg mL^{-1} was deposited atop the active layer at 3000 rpm for 30 s to afford a PDINO cathode buffer layer with thickness of *ca*. 10 nm. Finally, top Al electrode was deposited in vacuum onto the cathode buffer layer at a pressure of *ca*. 5.0×10^{-5} Pa. The typical active area of the devices is 4.7 mm^2.

Optical microscope (Olympus BX51) was used to define the active area of the devices. Oriel Sol3A Class AAA Solar Simulator (model, Newport 94023A) with a 450 W xenon lamp and an air mass (AM) 1.5 filter was used as the light source. The spectrum of IPCE was measured by Solar Cell Spectral Response Measurement System QE-R3-011 (Enli Technology Co., Ltd., Taiwan). The light intensity at each wavelength was calibrated with a standard single-crystal Si photovoltaic cell.

Spectro-electrochemical and chemical doping measurements. For spectro-electrochemical measurements, the measured compounds were dissolved in chloroform at the concentration of 10 mg mL^{-1} and the polymers were conducted by cast film onto an indium-tin oxide (ITO) coated glasses as working electrodes. To determine the chemical potential, we used the reference electrode of Ag/AgCl electrode and the auxiliary electrode of Pt. The oxidation and reduction measurements of the polymer

films were in the electrolyte of tetra-n-butylammonium hexafluorophosphate in acetonitrile (CH_3CN) at the concentration of 38.7 mg mL^{-1}. The absorption spectra were measured from the samples before and after charging in the spectral range from visible to near infrared. To quantitatively estimate the efficiency of iPP generation, we followed the chemical doping method used by Tautz et al.[7]. In the measurements, the polymer of N2200 was solved in anhydrous 1,2-Dichlorobenzene (Aladdin) with a concentration of 150 μg mL^{-1} and filled into a fused silica cell with a light pass of 2 mm. Tris-(pentafluorophenyl)borane (TCI) is a strong Lewis acid which can generate hole polarons in conjugated polymers. For chemical doping, 10 μL dilute solution of tris-(pentafluorophenyl)borane (60 μg mL^{-1}) was added into 290 μL N2200 solution. The absorption of the pure solution was measured by adding 10 μL solvent into 290 μL N2200 solution to ensure the same concentration of N2200.

Data simulation based on global fitting analysis. To further support the explanations of the experimental data, we simulate the experimental data of the neat acceptor and the blend films. For TA data recorded from the N2200 film, we used a model considering two parallel species representing iPPs and EXs. The spectral feature of iPPs is set to be the absorption spectrum of polarons derived by electrochemical doping measurements. For TA data recorded from the blend film, we adopted a model with three species. Two independently decaying species represent the iPPs and EXs and a rising component represents the states resulted from iPP-mediated hole transfer.

Supplementary References:

1. Becker, P.C., Fork, R.L., Cruz, C.H.B., Gordon, J.P. & Shank, C.V. Optical stark effect in organic dyes probed with optical pulses of 6-fs duration. *Phys. Rev. Lett.* **60**, 2462-2464 (1988).
2. Skotheim, T.A. & Reynolds, J.R. *Handbook of Conducting Polymers*, Third Edition edn. CRC Press (2007).
3. Herrmann, D. et al. Role of structural order and excess energy on ultrafast free charge generation in hybrid polythiophene/Si photovoltaics probed in real time by near-infrared broadband transient absorption. *J. Am. Chem. Soc.* **133**, 18220-18233 (2011).
4. Osterbacka, R., An, C.P., Jiang, X.M. & Vardeny, Z.V. Two-dimensional electronic excitations in self-assembled conjugated polymer nanocrystals. *Science* **287**, 839-842 (2000).
5. Sheng, C.X., Tong, M., Singh, S. & Vardeny, Z.V. Experimental determination of the charge/neutral branching ratio eta in the photoexcitation of pi-conjugated polymers by broadband ultrafast spectroscopy. *Phys. Rev. B* **75**, 085206 (2007).
6. De Sio, A. et al. Tracking the coherent generation of polaron pairs in conjugated polymers. *Nat. Commun.* **7**, 13742 (2016).
7. Tautz, R. et al. Structural correlations in the generation of polaron pairs in low-bandgap polymers for photovoltaics. *Nat. Commun.* **3**, 970 (2012).
8. Heeger, A.J., Kivelson, S., Schrieffer, J.R. & Su, W.P. Solitons in conducting polymers. *Rev. Mod. Phys.* **60**, 781-850 (1988).
9. Frisch, M.J. et al. *Gaussian 09 Revision D.01. Gaussian Inc. Wallingford CT 2013.*
10. Su, W.P., Schrieffer, J.R. & Heeger, A.J. Solitons in polyacetylene. *Phys. Rev. Lett.* **42**, 1698-1701 (1979).
11. Su, W.P., Schrieffer, J.R. & Heeger, A.J. Soliton excitations in polyacetylene. *Phys. Rev. B* **22**, 2099-2111 (1980).
12. Aghtar, M., Liebers, J., Struempfer, J., Schulten, K. & Kleinekathoefer, U. Juxtaposing density matrix and classical path-based wave packet dynamics. *J. Chem. Phys.* **136**, 214101 (2012).
13. Yao, Y., Qiu, Y. & Wu, C.-Q. Dissipative dynamics of charged polarons in organic molecules. *J. Phys. Conden. Matt.* **23**, 305401 (2011).
14. An, Z., Wu, C.Q. & Sun, X. Dynamics of photogenerated polarons in conjugated polymers. *Phys. Rev. Lett.* **93**, 216407 (2004).
15. Bredas, J.L. & Street, G.B. Polarons, bipolarons, and solitons in conducting polymers. *Acc. Chem. Res.* **18**, 309-315 (1985).
16. Bakulin, A.A., Hummelen, J.C., Pshenichnikov, M.S. & van Loosdrecht, P.H.M. Ultrafast hole-transfer dynamics in polymer/PCBM bulk heterojunctions. *Adv. Funct. Mater.* **20**, 1653-1660 (2010).
17. Kang, H. et al. From Fullerene-Polymer to All-Polymer Solar Cells: The Importance of Molecular Packing, Orientation, and Morphology Control. *Acc. Chem. Res.* **49**, 2424-2434 (2016).
18. Xue, L. et al. Naphthalenediimide-alt-Fused Thiophene D-A Copolymers for the Application as Acceptor in All-Polymer Solar Cells. *Chem. Asian J.* **11**, 2785-2791 (2016).
19. Xue, L. et al. Indacenodithienothiophene-naphthalene diimide copolymer as an acceptor for all-polymer solar cells. *J. Mater. Chem. A* **4**, 5810-5816 (2016).
20. Xue, L. et al. Synthesis and Characterization of Arylenevinylenearylene-Naphthalene Diimide Copolymers as Acceptor in All-Polymer Solar Cells. *J. Polym. Sci. Polym. Chem.* **55**, 1757-

1764 (2017).

21. Gao, L. et al. All-polymer solar cells based on absorption-complementary polymer donor and acceptor with high power conversion efficiency of 8.27%. *Adv. Mater.* **28**, 1884-1890 (2016).

22. Yan, H. et al. A high-mobility electron-transporting polymer for printed transistors. *Nature* **457**, 679 (2009).

23. Zhang, Z.-G. et al. Perylene diimides: a thickness-insensitive cathode interlayer for high performance polymer solar cells. *Energy Environ. Sci.* **7**, 1966-1973 (2014).
